# Quantity changes in acute headache medication use among patients with chronic migraine treated with eptinezumab: subanalysis of the PROMISE-2 study

**DOI:** 10.1186/s10194-022-01482-0

**Published:** 2022-09-06

**Authors:** Robert P. Cowan, Michael J. Marmura, Hans-Christoph Diener, Amaal J. Starling, Jack Schim, Joe Hirman, Thomas Brevig, Roger Cady

**Affiliations:** 1 Stanford Health Care, Palo Alto, CA USA; 2 Jefferson Headache Center, Philadelphia, PA USA; 3 Medical Faculty of the University Duisburg-Essen, Institute for Medical Informatics, Biometry and Epidemiology, Essen, Germany; 4 Mayo Clinic Arizona, Scottsdale, AZ USA; 5 The Neurology Center of Southern California, Carlsbad, CA USA; 6 Pacific Northwest Statistical Consulting, Inc., Woodinville, WA USA; 7 H. Lundbeck A/S, Valby, Denmark; 8 Lundbeck LLC, Deerfield, IL USA; 9 RK Consults, Ozark, MO USA; 10 Missouri State University, Springfield, MO USA

**Keywords:** Chronic migraine, Eptinezumab, Medication-overuse headache, Serotonin 5-HT_1_ receptor agonists, Analgesics

## Abstract

**Background:**

Patients with chronic migraine (CM) treated with eptinezumab in the PROMISE-2 trial achieved greater reductions in migraine and headache frequency, impact, and acute headache medication (AHM) use than did patients who received placebo. This post hoc analysis examines relationships between headache frequency reductions and changes in AHM use in patients in PROMISE-2.

**Methods:**

PROMISE-2 was a double-blind, placebo-controlled trial conducted in adults with CM. Patients were randomized to eptinezumab 100 mg, 300 mg, or placebo, administered intravenously once every 12 weeks for up to two doses. Patients recorded headache/AHM information daily and for each event in an electronic diary; data from all days with daily reports were included. Shifts in headache frequency and AHM use were assessed in the three populations: total CM population, patients with CM and medication-overuse headache (MOH), and patients with CM and MOH who were ≥ 50% responders during treatment (response over weeks 1–24).

**Results:**

A total of 1072 adults with CM received treatment (eptinezumab, *n* = 706; placebo, *n* = 366). Mean baseline headache frequency was 20.5 days; mean baseline AHM days was 13.4; 431 patients had MOH, of which 225 (52.2%) experienced ≥50% response over weeks 1–24. Relative to baseline, the proportion of days with both headache and AHM use decreased 25.1% (eptinezumab) versus 17.0% (placebo) in the total population (*N* = 1072), 29.2% versus 18.4% in the MOH subpopulation (*n* = 431), and 38.3% versus 31.5% in the CM with MOH population with ≥50% response subgroup (*n* = 225) during weeks 1–24. The proportion of days with headache and triptan use decreased 9.1% (eptinezumab) versus 5.8% (placebo), 11.8% versus 7.2%, and 14.5% versus 12.6%, respectively. Reductions in other AHM types were smaller.

**Conclusions:**

In this post hoc analysis, eptinezumab use in patients with CM was associated with greater decreases in days with headache with AHM overall and with triptans in particular. The magnitude of effect was greater in the subgroup of CM patients with MOH and ≥ 50% response.

**Trial registration:**

ClinicalTrials.gov Identifier: NCT02974153.

**Graphical abstract:**

Eptinezumab reduces headache frequency and acute medication use in patients with chronic migraine.
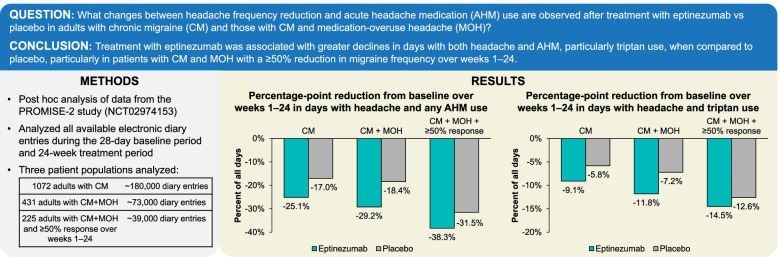

## Introduction

Acute headache medication (AHM) use is almost universal among individuals with chronic migraine (CM). It is estimated that > 90% of patients with migraine use some form of AHM [1–3]. In the large (*N* = 13,624) Chronic Migraine Epidemiology and Outcomes (CaMEO) study conducted in the US, nearly one-quarter (22.9%) of survey respondents indicated that they were current users of acute prescription migraine medications, with the most frequently utilized agents being triptans (47.2%), opioids (37.3%), nonsteroidal anti-inflammatory drugs (31.9%), and barbiturates (12.8%) [4]. Additionally, this survey identified that use of over-the-counter medications was high among both discontinued and current AHM users at 88.7% and 84.6%, respectively [4]. Use of AHM (especially sustained over time) can result in side effects, medical complications, and, in some patients, the development and persistence of medication-overuse headache (MOH); reduction of reliance on AHM is recognized as an important preventive treatment goal [5–15]. MOH is a secondary headache disorder commonly associated with CM, with higher rates of disease impact, medical cost, and disability [11].

Eptinezumab is indicated for the preventive treatment of episodic and chronic migraine in adults and is the only intravenously administered treatment out of several newer migraine-specific treatments for patients with migraine [12, 15–18]. PROMISE-2 was a pivotal phase 3, multicenter, randomized, double-blind, placebo-controlled evaluation of eptinezumab for the preventive treatment of CM [19]. Individuals who received eptinezumab (100 mg or 300 mg intravenously once every 12 weeks) in PROMISE-2 achieved greater reductions (≥50% or ≥ 75%) in migraine and headache frequency, impact, and AHM use than did patients who received placebo [19]. This post hoc analysis of data from PROMISE-2 was conducted to examine the relationships between headache frequency reduction and changes in AHM use in adults with CM, not only in the full PROMISE-2 study population, but also in the subpopulations of patients with the secondary disorder MOH and patients with MOH who were ≥ 50% responders over the course of treatment.

## Methods

### Study oversight

The protocol for PROMISE-2 was approved by the independent ethics committee or institutional review board for each study site, and the study was conducted in accordance with current Good Clinical Practices as referenced in the International Conference on Harmonisation of Technical Requirements for Registration of Pharmaceuticals for Human Use guidelines, the principles of the Declaration of Helsinki, and local regulatory requirements [19]. Written informed consent was obtained from all participants prior to study initiation [19]. This study is registered on ClinicalTrials.gov under the identifier NCT02974153 [19].

### Study design and patients

PROMISE-2 was a randomized, double-blind, placebo-controlled phase 3 study that evaluated the safety and efficacy of eptinezumab (100 mg or 300 mg) in adults (18–65 years of age, inclusive) with a greater than 12-month history of CM, the diagnosis of which was made using the International Classification of Headache Disorders, 3rd edition (beta) criteria (ICHD-3β) [19, 20]. A detailed description of the study design has been published [19].

Briefly, eligible patients received eptinezumab 100 mg, eptinezumab 300 mg, or placebo administered intravenously every 12 weeks for up to two doses (day 0 and week 12). Acute medication use was permitted throughout the study. In an effort to limit the enrollment of patients likely to be refractory to preventive treatment, barbiturate and prescription opioid use were limited during the run-in period to no more than 4 days/month, with the expectation that patients would continue limiting use to ≤4 days/month throughout the treatment period [19].

Patients with a dual diagnosis of CM and MOH were permitted in the PROMISE-2 study [19]. The diagnosis of MOH as a secondary headache disorder was made using the ICHD-3β diagnostic criteria [20] and was determined at the screening visit [21].

### Outcome measures

Throughout the screening and study periods, patients recorded information about their daily experiences (evening report; completed regardless of whether they had a headache) and during an event (i.e., per headache) in an electronic diary [19]. Data captured included headache episodes, migraine attacks, and AHM use, the latter of which was provided as a list of choices: ergotamine, triptan, simple analgesic, opioid, and combination analgesic.

### Statistical methods

Data from all days with completed electronic diary evening reports were included in these analyses. Shifts in AHM use were assessed in three populations: adults with CM (full study population), adults with CM and MOH (prospectively diagnosed subgroup), and adults with CM and MOH who were ≥ 50% responders over weeks 1–24. The latter subgroup was explored to confirm the relationship between headache day frequency and AHM use, as well as to explore differential shifts in AHM type with confirmed reduction in headache day frequency.

Reductions in monthly migraine days (MMDs) were evaluated by comparing baseline migraine frequency to frequency in 4-week intervals, which were combined to produce frequency over the entire study (weeks 1–24). Data for the eptinezumab arms (100 mg and 300 mg) were pooled, given that the dose levels demonstrated similar efficacy and safety in the total population [19] and subpopulation with MOH [21], and had similar efficacy in other subgroup analyses [22, 23].

The nature of the data capture made it possible to determine days where patients treated a headache and which days they used AHM without a headache. Hence, for both the screening (baseline) and treatment (post-baseline: weeks 1–24) periods, days were categorized into four groups defined by the presence or absence of headache and AHM use: headache with AHM use, headache with no AHM use, no headache with AHM use, and no headache with no AHM use. The distribution of AHM type on headache days with AHM use was also evaluated.

The statistical tools used for this post hoc analysis were descriptive in nature. Given that lack of pre-specification, claims of statistically significant/definitive conclusions are not made.

## Results

### Patients

A total of 1072 adults with CM (mean age, 40.5 years) participated in PROMISE-2 [19]. Of these, 706 received eptinezumab and 366 received placebo. Demographic and baseline clinical characteristics have been published and were generally consistent across treatment groups. Participants were predominantly white (91.0%) and female (88.2%) [19]. Mean baseline headache frequency in the total CM population was 20.5 days, with an average of 13.4 AHM days reported during the 28-day screening period [19].

A total of 431/1072 (40.2%) patients at screening were given a diagnosis of MOH by trained clinicians who were aided with a worksheet of MOH ICHD-3β criteria [20] (eptinezumab, *n* = 286; placebo, *n* = 145); of these 225/431 (52.2%) were ≥ 50% responders over weeks 1–24 (eptinezumab, *n* = 176/286, 61.5%; placebo, *n* = 49/145, 33.8%). Detailed demographics for the subgroup with CM and MOH have been reported [19, 21].

### Data availability

Data from all days with medication data (i.e., completed electronic diary evening reports) were included in these analyses. In the entire CM population, this comprised 28,064 study days (eptinezumab, 18,504 days; placebo, 9560 days) during the 28-day screening/baseline period and 151,022 study days (eptinezumab, 100,390 days; placebo, 50,632 days) during the post-baseline period (weeks 1–24). Data availability for each population is summarized in Table 1.

**Table 1:** Number of days with completed electronic diary evening reports, by population and time period

Population	Eptinezumab		Placebo	
	Baseline	Weeks 1–24	Baseline	Weeks 1–24
CM, *N* = 1072 (eptinezumab, *n* = 706; placebo *n* = 366)	18,504	100,390	9560	50,632
CM + MOH, *N* = 431 (eptinezumab, *n* = 286; placebo *n* = 145)	7500	41,113	3805	20,423
CM + MOH + ≥50% response, *N* = 225 (eptinezumab, *n* = 176; placebo, *n* = 49)	4652	25,855	1263	6850

*CM* chronic migraine, *MOH* medication-overuse headache

### Effects of treatment on headache days and AHM use

**Fig. 1:**
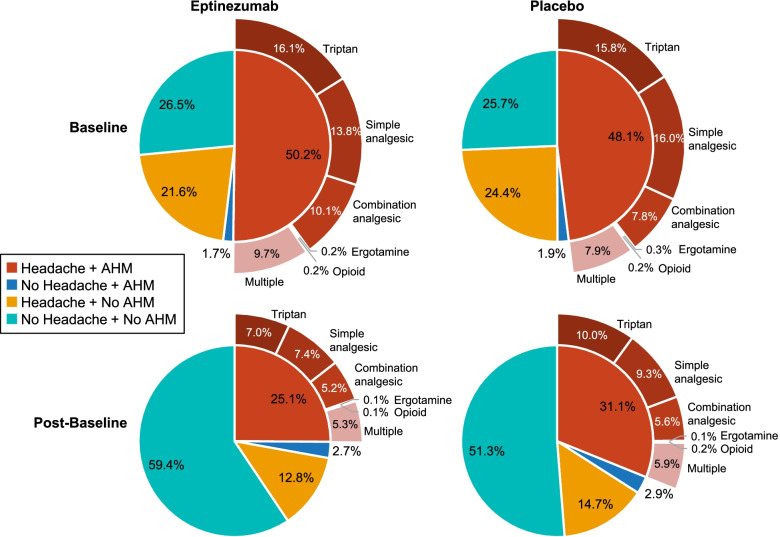
Days with AHM use among patients with CM, by headache status (with or without headache); AHM type included for days with headache and AHM use. AHM, acute headache medication; CM, chronic migraine

**Fig. 2:**
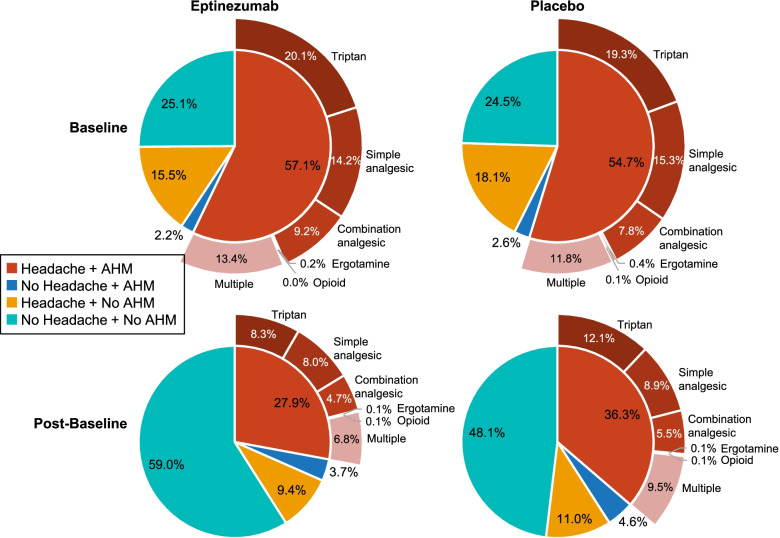
Days with AHM use among patients with CM and MOH, by headache status (with or without headache); AHM type included for days with headache and AHM use. AHM, acute headache medication; CM, chronic migraine; MOH, medication-overuse headache

**Fig. 3:**
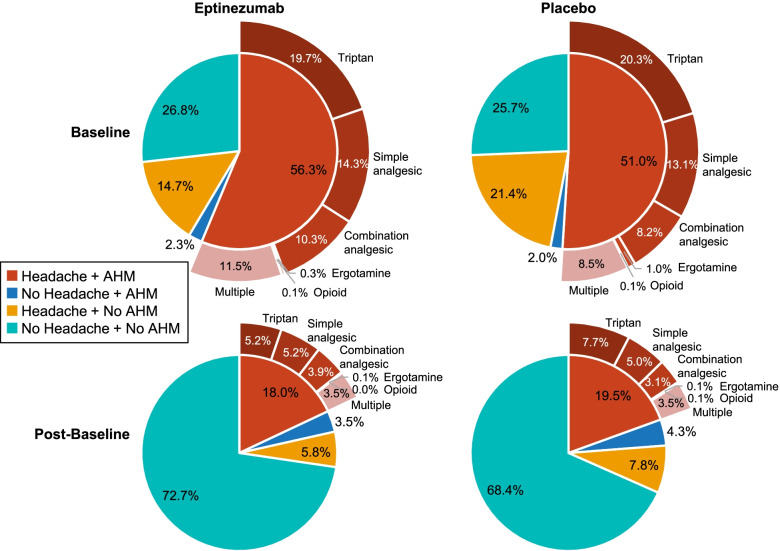
Days with AHM use among patients with CM and MOH who experienced ≥50% response over weeks 1–24, by headache status (with or without headache); AHM type included for days with headache and AHM use. AHM, acute headache medication; CM, chronic migraine; MOH, medication-overuse headache

Approximately half of the days during the baseline period (48.1–50.2%) were days on which the total population of patients with CM had a headache and used AHM (Fig. 1). In patients with CM and MOH (Fig. 2), including those with ≥50% response (Fig. 3), days with headache and AHM use comprised 51.0–57.1% of the baseline period. Interestingly, across populations, days with AHM use in the absence of headache were uncommon during baseline (1.7–4.6% of days).

Whereas post-baseline (week 1–24) reductions in the proportion of days with both headache and AHM were observed in both treatment groups in all three analysis populations, greater percentage-point reductions were consistently observed with eptinezumab compared to placebo: − 25.1% versus − 17.0%, respectively, in the entire CM population (Fig. 1); − 29.2% versus − 18.4% in the MOH subpopulation (Fig. 2); and − 38.3% versus − 31.5% in the ≥50% responder subgroup (Fig. 3). In patients with CM and MOH who experienced a ≥ 50% response over weeks 1–24, < 20% of the post-baseline period comprised headache days with AHM use.

In both the eptinezumab and placebo groups of all three analysis populations, the proportion of days with AHM use in the absence of headache was low at baseline and increased numerically only slightly during the post-baseline period. In the CM population, increases approximated 1.0% in both treatment groups. In the MOH subpopulation and ≥ 50% responder subgroup, increases in the proportion of days with no headache but with AHM use increased 1.2–1.5% with eptinezumab and 2.0–2.3% with placebo.

Reductions in the proportion of days with headache but no AHM use were observed in both treatment groups in all three analysis populations: − 8.8% versus − 9.7% in eptinezumab and placebo, respectively, in the entire CM population (Fig. 1); − 6.1% versus − 7.1% in the MOH subpopulation (Fig. 2); and − 8.9% versus − 13.6% in the ≥50% responder subgroup (Fig. 3).

In all three analysis populations, patients reported no headache and no AHM use for approximately one-fourth of days during the baseline period (Fig. 1). The proportion of days with no headache and no AHM use increased to 59.4% (eptinezumab) and 51.3% (placebo) during weeks 1–24 in the total CM population; to 59.0% and 48.1%, respectively, in the MOH subpopulation; and to 72.7% and 68.4%, respectively, in the MOH subpopulation with ≥50% response over weeks 1–24.

### Effects of treatment on AHM type

For days with both headache and AHM use, details regarding AHM type are provided in Fig. 1. In the total CM population, the proportion of days with headache and triptan use decreased 9.1% with eptinezumab and 5.8% with placebo over weeks 1–24 (Fig. 1). Reductions in headache days with triptan use were also observed in the MOH (eptinezumab, − 11.8%; placebo, − 7.2%; Fig. 2) and MOH with ≥50% response (eptinezumab, − 14.5%; placebo, − 12.6%; Fig. 3) subgroups. Smaller reductions in the proportion of headache days with combination analgesic use and headache days with simple analgesic use were also observed.

By limiting the analysis of AHM type to only those patients with headache and AHM use, it is evident that, in patients with CM, the most commonly utilized AHMs on headache days during the screening period were triptans (32.4%), simple analgesics (29.5%), and combination analgesics (18.8%). During weeks 1–24, AHM type shifted slightly to fewer triptan days (29.5%), more combination analgesic days (19.7%), and more days where multiple AHMs were used (baseline, 18.4%; post-baseline, 20.2%).

**Fig. 4:**
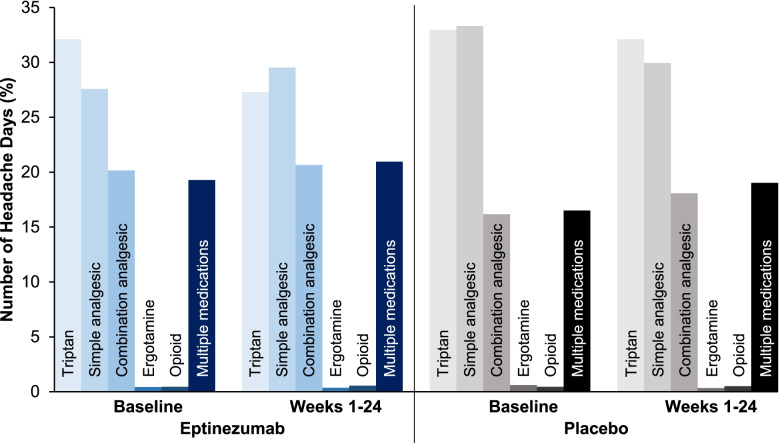
Acute headache medication type on headache days with acute headache medication use at baseline and weeks 1–24 in patients with CM who received eptinezumab and placebo. CM, chronic migraine

There were slight differences in the shifts in the eptinezumab and placebo groups (Fig. 4), with an apparent shift from triptans to simple and combination analgesics among patients who received eptinezumab, but with the reduction in AHM use in the placebo group being spread across triptans and simple analgesics (with increased combination analgesic use).

## Discussion

In this post hoc analysis of data from the PROMISE-2 study, eptinezumab use in patients with CM was associated with greater declines in days with both headache and AHM, particularly triptan use, when compared to placebo. The magnitude of effect was greatest in the subgroup of patients with CM and MOH who experienced ≥50% response (comprising almost twice as many patients treated with eptinezumab than patients receiving placebo), suggesting that preventive treatment with eptinezumab can result in decreased AHM use. This is consistent with observed patterns of AHM use in that a direct relationship between headache frequency and AHM use exists. Evidence from the placebo groups of each analysis support this relationship; that is, as with active treatment, reductions in headache frequency in the placebo group were associated with reductions in AHM use [4, 11, 24]. The placebo response observed in this analysis may have been related to several factors, such as patient expectations, frequent interactions with study personnel, higher likelihood of receiving active treatment in the study (2:1), and invasiveness of receiving study drug (i.e., intravenous administration) [25–29].

Interestingly, reductions in the proportion of days with headache but no AHM use were observed in both treatment groups in all three analysis populations, suggesting that patients were better able to optimize AHM use even on headache days, for instance, possibly due to milder, more manageable symptoms or increased availability of prescription AHM due to lower headache frequency. A previous analysis has demonstrated a reduction in the proportion of headache with severe pain following eptinezumab vs placebo treatment [30]. The pronounced reduction in triptan use is consistent with this hypothesis; that is, patients who, before preventive treatment, required triptans to manage symptoms were, after effective preventive treatment, able to manage relatively more headaches with simple analgesics. However, there were slight increases in the proportions of each analysis group with no headache but with AHM use, a finding that suggests that factors unrelated to headache frequency may contribute to the decision to use AHM in some patients.

The potential clinical benefits of the observed decline in AHM use are considerable, such as the opportunity to reduce medical complications. Central nervous system and gastrointestinal-related side effects are common across the AHM classes [5]. Triptan users may also experience non-cardiac chest discomfort, paresthesias, and skeletal pain [5, 6], which could lead to non-adherence with prescribed regimens. Headache medication intake can also contribute to acute and chronic kidney injury [31, 32] and ischemic complications [33]. Thus, a potential benefit of reduced AHM use is reduced adverse event occurrence and medical cost, leading to improved quality of life. Furthermore, because the costs of prescription AHMs can be a major contributor to overall direct and migraine-related medication costs (in the US), there is potential financial benefit for both patients and payors that should be considered [24, 34, 35].

It is also important to keep in mind that patients with migraine who use AHM, regardless overuse, have greater prevalence of cardiovascular and gastrointestinal comorbidities, depression, and anxiety and demonstrate higher levels of disability and functional decline than do patients not currently using AHM [4, 35]. While it may be tempting to surmise that reductions in AHM use might reduce these disorders and impairments, there are other factors that may influence their occurrence, including headache frequency, lifestyle-related risk factors, and genetic susceptibility [36–39].

It was previously shown that, in patients with CM and MOH in PROMISE-2, 29% of patients who received eptinezumab versus 6.3% of patients who received placebo dropped and remained below diagnostic thresholds for both CM and MOH over the 24-week treatment period [40]. In patients with CM and MOH in this post hoc analysis, 59% of the 24-week post-baseline period comprised days without headache or AHM use for patients treated with eptinezumab compared with 48% for patients receiving placebo. Although the implications of these changes are incompletely understood, it is possible they could also affect changes in the occurrence/persistence of concomitant mood disorders and in the utilization of healthcare resources, both of which are increased in patients with CM and MOH [34, 41–45].

The prevailing stigma [46–48] facing patients with CM and MOH is iatrogenic; that is, MOH is an illness caused by a patient’s overuse of their acute medications. Some older studies have suggested that anxiety related to anticipation of headache is associated with increased AHM use [49–51]; however, other studies have suggested that other factors are more strongly associated with higher AHM use (eg, number of headache days) [44, 52] or have not documented that the patients were taking medication in anticipation of an attack [48, 53]. In the current analysis, the proportion of days without headache but with AHM use was consistently small (< 5% of days) across populations analyzed, with minor increases during weeks 1–24 possibly attributed to non–headache-related uses of acute medication, such as myofascial and other pain disorders [54]. Results of these analyses suggest that when headache frequency is controlled, so too is the frequency of acute medication use. This is underscored by the fact that patients in the PROMISE-2 study with MOH were not provided any MOH-specific treatment intervention (such as education or a directive to stop use) and that those with MOH who experienced a sustained clinical response (i.e., a ≥ 50% reduction in migraine frequency over 24 weeks) had the largest reductions in days with AHM use compared to the total CM and total MOH populations in both treatment groups.

Although PROMISE-2 enrolled patients with CM, a relationship between ineffective AHM use and progression from episodic migraine (EM) to CM has been reported [55]. Thus, another potential benefit of eptinezumab that warrants further study is reduced headache frequency and AHM use in patients with EM. Additionally, previous work has suggested that in patients with EM and MOH, the rate of MOH depends highly on the AHM type, with triptan- and ergot-related MOH occurring less frequently compared to analgesic- and opioid-related MOH [10]. In contrast, the results presented here showed eptinezumab’s pronounced effect on patients with CM taking triptan medications. Future work would help clarify how eptinezumab-linked reductions in AHM use in patients with EM are influenced by AHM type, and if the baseline distributions of AHM type are similar to those of PROMISE-2.

### Limitations

The findings reported here have limitations, including the post hoc nature of the analysis and the smaller size of the CM + MOH and CM + MOH + ≥50% response subpopulations compared to the total population. Medication overuse was quantified based on protocol-defined post-baseline intervals, in which 12- and 24-week results (i.e., the number of days patients used acute migraine medication during these timepoints) were calculated by taking the averages of smaller, 4-week increments. Other study protocols have used benchmarks of 1 year [56], for instance, to quantify meaningful changes in medication overuse; these differences in study design reflect the overall challenge with quantifying (and thus treating) medication overuse [57]. However, PROMISE-2 was placebo-controlled for 6 months. The population of PROMISE-2 was limited to individuals with CM; those with EM or other headache disorders were excluded from participation. Thus, findings may not be generalizable to these latter conditions. Patients using opioids or barbiturates on 4 or more days during screening were not included in PROMISE-2, so the MOH subgroup did not include patients with overuse of those classes of AHM alone. Opioid and barbiturate use was limited in the PROMISE-2 study because many headache specialists believe high use often makes the patient refractory to preventive treatment [19] and may be indicative of a substance use disorder. Opioid and barbiturate use is less common outside of the United States [58], thus results of PROMISE-2 could be more generalizable to other countries. However, an ad hoc study may be necessary given that one-third of people in the United States who have migraine use opioids or barbiturates as an acute treatment [19]. Finally, this analysis only examined the relationship between AHM use and headache frequency, not other factors that could have influenced AHM use, such as the patients’ most bothersome symptom, symptom severity, and headache impact.

## Conclusion

Daily eDiary entries in the PROMISE-2 study—over 177,000 in total—were able to capture in detail the number of days patients experienced headache, the number of days of AHM use, and AHM type. Eptinezumab treatment in patients with CM was associated with greater decreases in headache days associated with AHM (particularly triptan) use compared to placebo in this post hoc analysis of PROMISE-2. Similarly in patients with CM and MOH, and most notably in the subset with ≥50% response, the magnitude of reduction in days with both headache and AHM use was greater with eptinezumab than placebo.

## Data Availability

In accordance with EFPIA’s and PhRMA’s “Principles for Responsible Clinical Trial Data Sharing” guidelines, Lundbeck is committed to responsible sharing of clinical trial data in a manner that is consistent with safeguarding the privacy of patients, respecting the integrity of national regulatory systems, and protecting the intellectual property of the sponsor. The protection of intellectual property ensures continued research and innovation in the pharmaceutical industry. Deidentified data are available to those whose request has been reviewed and approved through an application submitted to https://www.lundbeck.com/global/our-science/clinical-data-sharing.

## Abbreviations

AHM: Acute headache medication
CM: Chronic migraine
EM: Episodic migraine
MMD: Monthly migraine days
MOH: Medication-overuse headache
